# Export of ice nucleating particles from a watershed

**DOI:** 10.1098/rsos.170213

**Published:** 2017-08-30

**Authors:** Jarl Are Larsen, Franz Conen, Christine Alewell

**Affiliations:** Department of Environmental Sciences, University of Basel, Bernoullistrasse 30, Basel, 4056, Switzerland

**Keywords:** ice nucleating particles, production rate, stability, watershed

## Abstract

Ice nucleating particles (INP) active at a few degrees below 0°C are produced by a range of organisms and released into the environment. They may affect cloud properties and precipitation when becoming airborne. So far, our knowledge about sources of biological INP is based on grab samples of vegetation, soil or water studied in the laboratory. By combining measurements of INP concentrations in river water with river water discharge rates over the course of 16 months, we obtained a lower limit for the production rate of INP in a watershed covering most of Switzerland (4 × 10^5^ INP_−8_ m^−2^ d^−1^). Coincidentally, we found that INP_−8_ are likely to retain their potential for catalysing ice formation in the natural environment for at least several months before they are mobilized by an intensive rainfall, washed into the river and exported from the watershed.

## 1. Introduction

Without a catalysing surface, water remains liquid in a supercooled state until cooled below about −37°C. Some organisms form at their surface macromolecules, which facilitate ice formation at temperatures just a few degrees below 0°C. Examples include bacteria dwelling on plant leaves and transforming dew or rain droplets into ice crystals. Sudden growth of an ice crystal can damage a leaf surface and open bacterial access to plant nutrients [1]. Lichens may benefit by early ice formation from a greater acquisition of water due to the lower vapour pressure above ice, compared with liquid water [2,3]. Fungi catalysing ice formation in soil can thereby cleave soil aggregates as well as decrease bulk density and thereby gain access to otherwise inaccessible resources [4]. Laboratory experiments have shown that biological ice nucleation is mediated by macromolecules, which can be released from the organism that produced them and still retain their specific property [4,5]. These small ice nucleating particles (INP) are then dispersed in the environment and form ice wherever appropriate moisture and temperature conditions are encountered. Clouds consisting of liquid water droplets at temperatures below 0°C are such environments. Of particular interest are INP active at −8°C or warmer (INP_−8_). Between −3°C and −8°C riming and splinter production can lead to ice multiplication [6], so that a small number of INP_−8_ (less than 10 m^−3^) can have a large impact on cloud glaciation [7].

Ice formation is the initial step by which precipitation can be triggered [8]. Accordingly, there is a long record of biological INP studies in atmospheric science. An early discovery was that decaying leaf litter is a likely source of very small and efficient INP [9–12], which had earlier been found in hailstones [13] and other precipitation samples [14]. The role of clouds in the context of climate change has renewed interest in biological INP [15]. Recent studies provide increasing evidence for the variety of sources, properties and concentrations of biological IN-active macromolecules [16–19]. However, experimental challenges have so far precluded an estimate of the rate at which such INP may be produced in the natural environment at larger scales (e.g. landscape scale). Here, we report on an unconventional approach to estimate the lower limit of an INP_−8_ production rate by measuring the export rate of INP with river water from a well-constrained watershed. From the analysis of its temporal pattern, we simultaneously obtain an estimate for how long macromolecular INP can retain their activity in the natural environment.

## 2. Methods

Water was sampled weekly, with few interruptions, from the river Rhine in Basel, Switzerland (47°33′44.3′′ N, 7°35′14.9′′ E, 250 m.a.s.l.) over a period of 16 months, from 10 June 2015 until 20 October 2016. Each sample (40 ml) was collected with a sterile polypropylene tube from a cable (reaction) ferry in the middle of the river (Klingentalfähre ‘Vogel Gryff’), taking care to avoid contamination from the hull and avoiding sampling when other boats were in the upstream vicinity. The samples were filtered through 5.0 and 0.22 µm syringe filters. From each filtrate, we prepared 52 aliquots of 100 µl in 0.5 ml Eppendorf tubes and analysed them within one hour after sampling. For analysis, the tubes were cooled in a cold bath at a rate of 0.3°C min^−1^ from −2 to −10°C. Freezing was detected automatically by the decrease in light transmission through the vertical axis of each tube as recorded by a camera installed above them. Four Pt1000 sensors recorded the concurrent freezing temperature. A detailed description of the apparatus can be found in Stopelli *et al*. [20]. When INP concentrations were so high that all tubes froze before reaching −10°C, we analysed 1 : 10 or 1 : 100 dilutions of the sampled water. The Milli-Q water used for dilution was tested and found to be free of INP active in the temperature range examined in this study.

**Figure 1.**
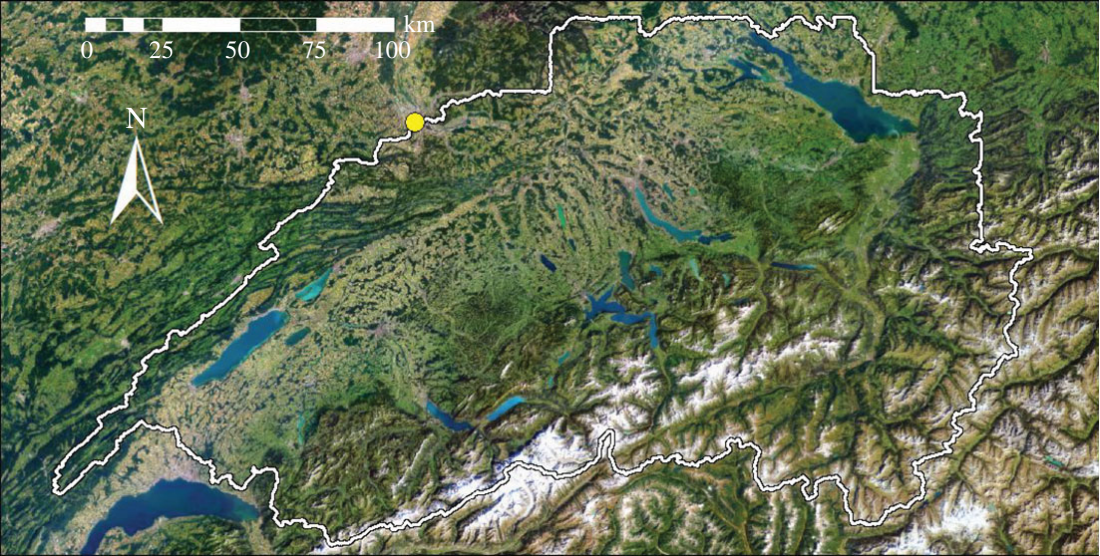
Satellite image of the watershed (border in white) with a yellow circle marking the sampling point in Basel (source: Landsat25/Swisstopo).

Data on river discharge were provided by the Federal Office for the Environment (FOEN) and turbidity data by the Department of Environment, Canton Basel-Stadt. Daily liquid precipitation for the watershed was calculated using spatially distributed precipitation and 2 m air temperature data (RhiresD and TabsD, supplied by Meteoswiss), using a threshold temperature of 0°C to distinguish between liquid and solid precipitation. The total watershed of the river Rhine upstream of Basel is approximately 3.6 × 10^4^ km^2^ (calculated from the digital elevation model Swisstopo DHM25). Most of it is covered with agricultural fields (24%), grassland (21%) or forest (30%) (Corine Land Cover, 2012). Higher elevations are covered by alpine meadows, rocks, snowfields and glaciers (figure 1).

## 3. Results and discussion

**Figure 2.**
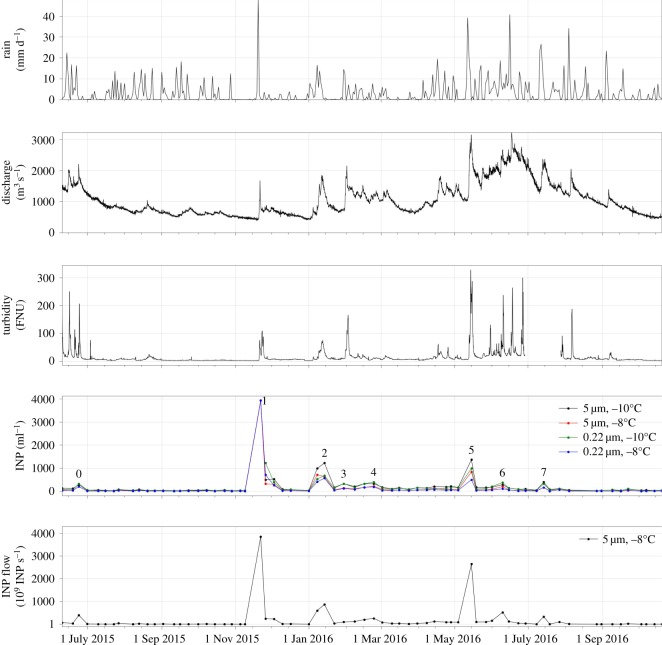
Time series of (from top to bottom): (1) average daily rain for the watershed (mm d^−1^), (2) water discharge at Basel (m^3^ s^−1^), (3) turbidity (Formazin Nephelometric Units), (4) measured INP (ml^−1^) (peaks identified with numbers 0–7 are used for the correlation in figure 3) and (5) INP flow (INP s^−1^).

The watershed received liquid precipitation in most weeks of the observation period. Longer spells without rain occurred in November 2015, and in February and March 2016 (figure 2). Rainfall exceeding 15 mm d^−1^ led within 1–2 days to a marked increase in river discharge at the sampling point in Basel and was accompanied by greater turbidity. Larger discharge rates in summer 2016 resulted from a combination of more frequent rainfall and snowmelt in the upper parts of the watershed. Concentrations of INP_−8_ were clearly elevated during peaks in water discharge (note: a peak discharge at the beginning of August 2016 was not sampled for INP). Values during base flow remained below 100 INP_−8_ ml^–1^. The mean onset of freezing was at −5.5°C. The highest onset of freezing was at –4°C (24 February 2016, 10 March 2016, 31 March 2016, 28 April 2016) and the lowest at −7.5°C (15 October 2015). On average, 79% of INP_−8_ that had passed the 5.0 µm filter also passed the 0.22 µm filter. Moffett [21] reported INP_−7_ concentrations between about 300 and 500 ml^−1^ for a river transect in Wales (UK), similar to some of the peak concentrations we have seen in the Rhine. Between 33 and 68% of these INP_−7_ were smaller than 0.22 µm. His sampling of the upper part of the river transect, where the higher concentrations were found, was conducted after a rainfall event (9.9 mm daily total precipitation in the region on the preceding day, UK Met Office, HadUKP) that led to a doubling of the discharge in the river (data from the National River Flow Archive, NRFA).

The export rate of INP_−8_ from the watershed upstream of Basel was calculated assuming a homogeneous mixture of INP in the river water (figure 2). The time series of the export rate resembles that of the INP concentration regarding the timing of peaks and their relative size. An exception is the peak in the middle of May 2016, which, due to the large snowmelt-driven flow, shows a relatively large INP flow compared with the INP concentration at the same time.

If INP_−8_ in river water would originate mainly from fresh water organisms, their concentration in Basel would be high during baseflow, and low during peak flow. The former, because a large fraction of baseflow water had a long residence time in lakes upstream, where it should have become enriched in INP_−8_, if fresh water organisms were their source. The latter, because additional rain water during peak flow would dilute INP_−8_ concentrations in the baseflow, as most of the precipitation events in the watershed have concentrations less than 10 INP_−8_ ml^−1^ [22]. Neither is the case. Although some of the INP_−8_ are probably produced in the freshwater system [23,24], our data strongly suggest that the majority of INP_−8_ in Rhine water at Basel are transported by surface or subsurface flow to the freshwater system. Most likely sources of the INP_−8_ are plant surfaces [25], plant litter [9,26] and soil [4,17,18], with only smaller contributions of wet fallout deposition due to the rain events (wash-out from the atmosphere). On or near their sources, INP_−8_ are likely to accumulate during periods with no or little rainfall. They are mobilized intermittently by intense rain and flushed into the river where they are discharged from the watershed during peak flow events. Production rate of INP_−8_ within the watershed must be much steadier than their export rate from it. Hence, the best estimate for a lower limit of the long term (16 months) production rate of INP_−8_ within the watershed rate is calculated as the mean of all observed INP discharge rates (INP_−8_ s^−1^) divided by the surface area of the watershed (3.6 × 10^10^ m^2^). The value of this estimate is 5 INP_−8_ m^−2^ s^−1^, or about 4 × 10^5^ m^−2^ d^−1^ (it does not make sense to estimate the uncertainty of this value from the distribution of individual export rates, because their value depends largely on temporal variations in peak flow events, not the production rate of the INP sources outside the freshwater). The estimate of an export rate of 4 × 10^5^ INP_−8_ m^−2^ d^−1^ is the minimum of a production rate because not all INP_−8_ produced on land surfaces in the watershed are necessarily exported by the river. Some will be aerosolized, a large fraction might remain in soil and eventually be mineralized, fed on, sorbed, sedimented or humified as any other fraction of soil organic matter. An unknown fraction will be de-activated before or during its transport in the river. The inventory of INP_−8_ in the boundary layer (lower approx. 1000 m of the atmosphere) above the watershed is relatively small, compared with the INP production rate. Analyses of fine dust collected throughout a year on a mountain station within the watershed (06°58′45′′ E, 47°02′58′′ N, 1136 m.a.s.l.) showed average concentrations around 10 INP_−8_ m^−3^ [27]. Hence, the boundary layer inventory of INP_−8_ (10 m^−3^ × 1000 m = 10^4^ m^−2^) is equivalent to 2.5% of the INP_−8_ produced every day (4 × 10^5^ INP_−8_ m^−2^).

**Figure 3.**
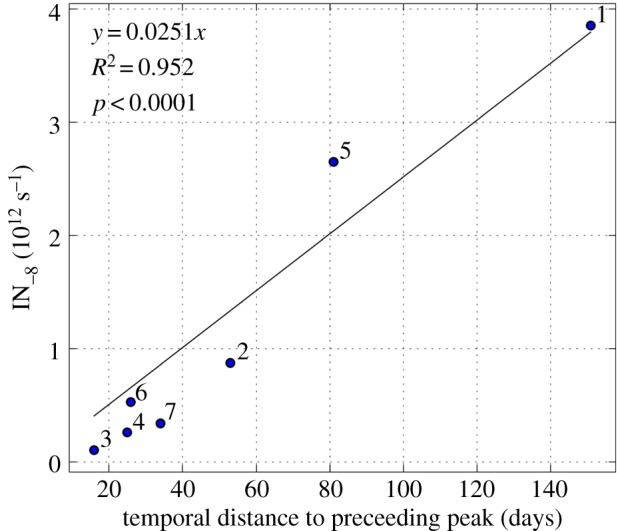
Linear correlation between number flow rate of INP_−8_ (10^12^ s^−1^) during peaks and temporal distance to the preceding INP_−8_ peak (days). Individual peaks are marked with numbers for comparison with figure 2.

Coincidentally, our data give an indication of the stability of INP_−8_ in the natural environment. The amount of INP_−8_ exported from the watershed during peak events correlated strongly (*R*_2_ = 0.952, *p* < 0.0001) with the temporal distance to the preceding INP peak (figure 3). This observation suggests that accumulated INP_−8_ indeed accumulate, and remain potentially active for many months. If ice nucleation activity was not stable, the correlation in figure 3 would not increase linearly over 140 days, but approach a plateau.

## 4. Conclusion

Sub-micrometre INP_−8_ production on land surfaces in a temperate climate is at least of the order of 10^5^–10^6^ m^−2^ d^−1^. The ice nucleating activity of these INP is stable over many months. They accumulate until mobilized by intermittent rain events and transported over longer distances. Export rates from a watershed can be measured easily by monitoring water discharge and INP concentrations in river water. Passage of a weather front with rain and strong winds may have a similar mobilizing effect on INP and their transport in air. However, an airshed has no confluence and is therefore much more difficult to survey. In the meantime, surveys of watersheds in different climatic regions may offer a glance into the likely strength of biological INP sources on larger scales than achieved before.

## Supplementary Material

Measured INP concentrations

## Data accessibility

The original data supporting this article are available on Dryad (http://dx.doi.org/10.5061/dryad.v31k1) [28].
